# Neurological management and work-up of neurotoxicity associated with CAR T cell therapy

**DOI:** 10.1186/s42466-021-00166-5

**Published:** 2022-01-10

**Authors:** Nora Möhn, Viktoria Bonda, Lea Grote-Levi, Victoria Panagiota, Tabea Fröhlich, Christian Schultze-Florey, Mike P. Wattjes, Gernot Beutel, Matthias Eder, Sascha David, Sonja Körner, Günter Höglinger, Martin Stangel, Arnold Ganser, Christian Koenecke, Thomas Skripuletz

**Affiliations:** 1 Department of Neurology, Hannover Medical School, Carl-Neuberg-Straße 1, 30625 Hannover, Germany; 2 Department of Hematology, Hemostasis, Oncology and Stem Cell Transplantation, Hannover Medical School, Carl-Neuberg-Straße 1, 30625 Hannover, Germany; 3 Department of Diagnostic and Interventional Neuroradiology, Hannover Medical School, Carl-Neuberg-Straße 1, 30625 Hannover, Germany; 4 Department of Nephrology, Hannover Medical School, Carl-Neuberg-Straße 1, 30625 Hannover, Germany; 5 Institute of Intensive Care Medicine, University Hospital Zurich, Rämistrasse 100, 8091 Zürich, Switzerland

**Keywords:** Autoimmunity, Neurotoxicity, Immunotherapy, Chimeric Antigen Receptors, T-Lymphocytes

## Abstract

**Introduction:**

Treatment with CD19 chimeric antigen receptor (CAR) T cells is an innovative therapeutic approach for patients with relapsed/refractory diffuse large B cell lymphoma (r/rDLBCL) and B-lineage acute lymphoblastic leukemia (r/rALL). However, convincing therapeutic response rates can be accompanied by cytokine release syndrome (CRS) and severe neurotoxicity termed immune effector cell-associated neurotoxicity syndrome (ICANS).

**Methods:**

Single center, prospective observational study of fifteen consecutive r/r DLBCL patients treated with Tisagenlecleucel within 1 year at Hannover Medical School. Extensive neurological work-up prior to CAR T cell infusion included clinical examination, cognitive testing (Montreal-Cognitive-Assessment), brain MRI, electroencephalogram, electroneurography, and analysis of cerebrospinal fluid. After CAR T cell infusion, patients were neurologically examined for 10 consecutive days. Afterwards, all patients were assessed at least once a week.

**Results:**

ICANS occurred in 4/15 patients (27%) within 6 days (4–6 days) after CAR T cell infusion. Patients with ICANS grade 2 (n = 3) exhibited similar neurological symptoms including apraxia, expressive aphasia, disorientation, and hallucinations, while brain MRI was inconspicuous in either case. Treatment with dexamethasone rapidly resolved the clinical symptoms in all three patients. Regarding baseline parameters prior to CAR T cell treatment, patients with and without ICANS did not differ.

**Conclusions:**

In our cohort, ICANS occurred in only every fourth patient and rather low grade neurotoxicity was found during daily examination. Our results demonstrate that a structured neurological baseline examination and close monitoring are helpful to detect CAR T cell related neurotoxicity already at an early stage and to potentially prevent higher grade neurotoxicity.

**Supplementary Information:**

The online version contains supplementary material available at 10.1186/s42466-021-00166-5.

## Background

Therapy with chimeric antigen receptor (CAR)-T cells directed against the B-cell surface marker CD19 is a new therapeutic approach for patients with malignant B-cell diseases [3, 14, 24]. In a retrospective study of 523 patients with refractory DLBCL, only 7% of patients had a complete response after standard therapy and the median survival was 6.3 months [5]. In contrast, CAR T cell therapies showed significantly better response rates in their pivotal studies. Tisagenlecleucel exhibited an objective response rate (ORR) of 52% and a complete remission (CR) rate of 40% in patients with refractory or relapsed DLBCL [1]. Axicabatagene Ciloleucel for its part showed ORR and CR rates of 74% and 54%, respectively [14]. However, during establishment of CAR T cell-based therapies it was observed that the occurring side effects differed significantly from those under classical chemotherapy or antibody therapies. In particular, two characteristic clinical manifestations due to drug toxicity have been described: (1) Depending on the CD19 CAR T product, 58–94% of patients developed a cytokine release syndrome (CRS) [9, 15] triggered by a pronounced cytokine release from activated T cells in peripheral blood and consecutive systemic inflammation [28]. (2) Neurological adverse events termed immune effector cell associated neurotoxicity syndrome (ICANS) have been reported. The prevalence and severity of neurotoxicity depend on various factors such as previous therapies, tumor load, different designs of CAR T cells, CAR T cell dosage, and infusion regime [7, 8, 20, 22]. Although CRS and neurotoxicity are considered as separate CAR T cell-associated toxicities, as they do not occur simultaneously and are treated differently, there is evidence that CRS plays a role in the development of neurotoxicity and that the occurrence of severe CRS increases the risk of neurotoxicity [8]. ICANS can manifest with multiple and initially in unspecific symptoms such as tremor, headache, or neurocognitive deficits [16]. In severe cases, ICANS can lead to the development of brain edema with accompanying focal neurological deficits such as apraxia and dysarthria [16]. However, motor aphasia, seizures, and/or loss of consciousness might even occur without signs of brain edema [22]. Deaths due to neurotoxicity have been reported in 3% of patients [8, 14]. A specific therapy of ICANS is currently not available and thus corticosteroids were used in most studies as first-line therapy [2, 18]. There is still limited knowledge about the occurrence and description of ICANS symptoms and their temporal evolution. Here, we provide the results of extensive neurological monitoring of 15 adult DLBCL patients who received CAR T cell therapy in a prospective single center study. The aim of the study was to determine the frequency and severity of neurotoxicity in the local cohort and to develop a suitable diagnostic and therapeutic approach to minimize severe neurotoxicity.

## Methods

### Study design and setting

**Fig. 1:**
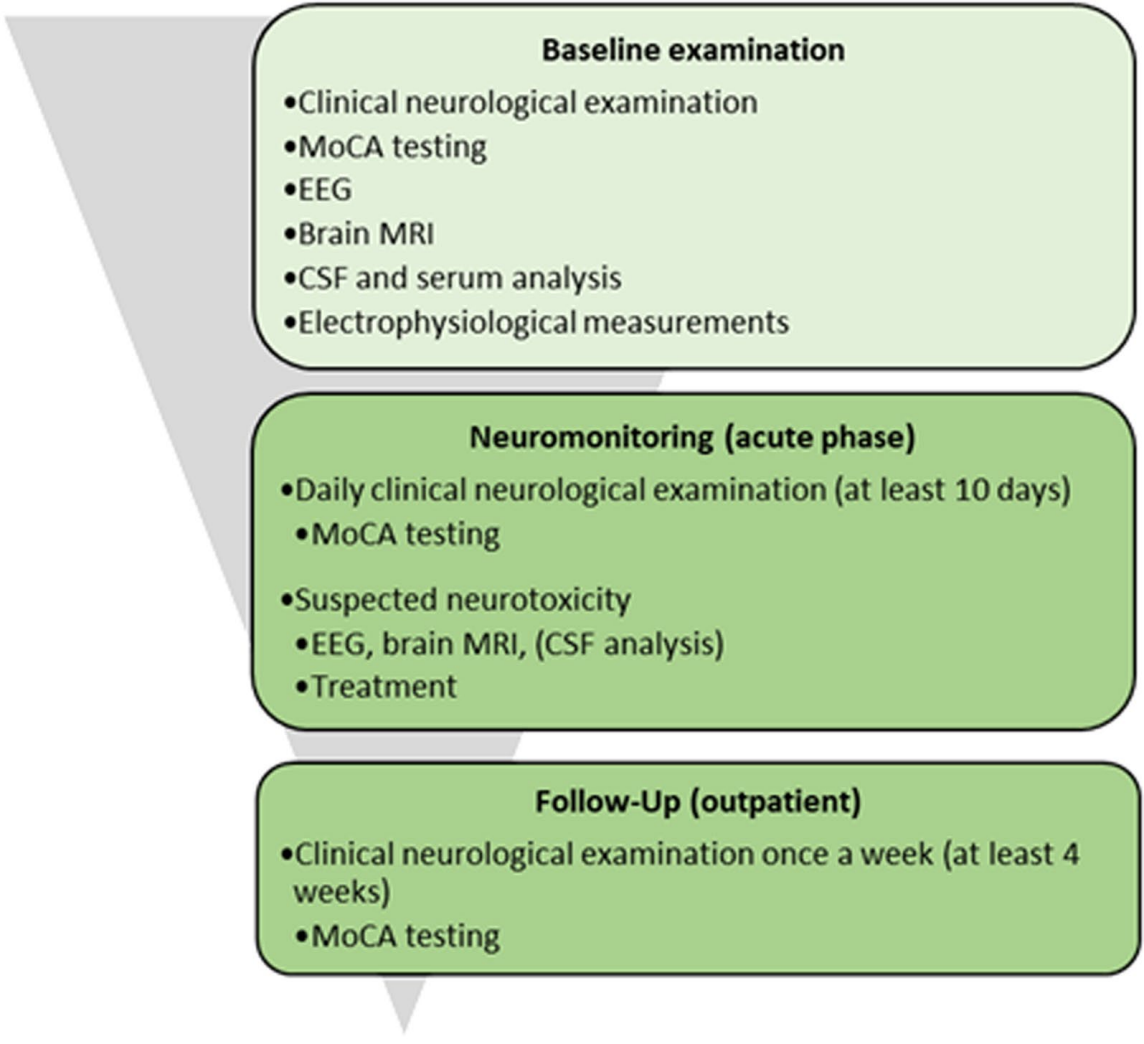
Hannover cohort neurological examination and monitoring before, during and after CAR T cell treatment. CSF: cerebrospinal fluid; EEG: electroencephalography; MoCA: Montreal Cognitive Assessment; MRI: magnetic resonance imaging

Eighteen patients with relapsed and refractory DLBCL were screened for treatment with Tisagenlecleucel between April 2019 and February 2020 and included into this prospective single center study at Hannover Medical School, Hannover, Germany. Fifteen patients were finally treated. All patients received an extensive neurological examination prior to CAR T cell therapy (Fig. 1). After CAR T cell infusion, patients were neurologically examined for 10 consecutive days. Up to 4 weeks following infusion, the patients were assessed at least once a week. Three patients had been screened but were not treated with CAR T cells. In two of them, the therapeutic concept was changed to palliative care and one patient received rituximab, bendamustine, and polatuzumab instead of CAR T cell therapy due to rapid lymphoma progression.

### Participants

Demographic data included age, sex, underlying disease, number of previous therapy regimes, CAR T cell dose, and time since diagnosis of DLBCL (see Additional file 1). Besides clinical neurological examination and MoCA testing (see Additional file 2), diagnostic procedures included brain MRI, electroencephalography (EEG), electroneurography (ENG) (median, peroneal and sural nerve), CSF examination (cell count, protein levels, albumin ratio, lactate, intrathecal immunoglobulin synthesis, oligoclonal bands (OCB)), and serum analysis for antineuronal antibodies. All patients gave written informed consent for participation (Hannover Medical School, 2413-2014). One patient included in the present study was previously described in detail [26].

### Diagnostic procedures

All patients received an extensive neurological monitoring before, during and after CAR T cell therapy. MoCA testing was used to detect cognitive deficits. For the first 10 days after CAR T cell treatment MoCA testing was applied daily, then once a week for at least 1 month. A total of three different versions of the MoCA test were applied. The ICANS grading was based on the current ASTCT consensus criteria [13].

All patients were examined by brain magnetic resonance imaging (MRI) using a whole body MR system operating at 3 Tesla (Skyra, Siemens, Erlangen, Germany). The multisequence protocol included 3D fluid attenuated inversion recovery (FLAIR), axial T2-weighted, diffusion-weighted imaging (DWI), susceptibility weighted imaging (SWI) and T1-weighted sequences before and after single dose intravenous Gadolinium administration. All images were analyzed by an experiences neuroradiologist (> 15 years) with special expertise in the field of neuroinflammation.

Electrophysiological diagnostics were performed with superficial stimulators and recording electrodes as conventional standard routine procedures [27]. For EEGs, electrode placement was performed according to the 10/20 system [11]. Besides hyperventilation with subsequent resting derivation, photostimulation with a flickering light was performed as well.

CSF and serum samples were analyzed by routine methods which have been described before [25]. Immediately after CSF withdrawal by lumbar puncture, CSF cell count, total CSF protein, and CSF lactate were analyzed. For further analyses the residual CSF was centrifuged (145 g for 15 min) and the supernatant frozen at − 70° C. CSF oligoclonal bands were determined by isoelectric focusing in polyacrylamide gels with consecutive silver staining [10].

Commercially available cerebellum primate slides (INOVA Diagnostics) and immunoblots with recombinant antigens (PNS-Blot, Ravo Diagnostika) were utilized for detection of antineuronal antibodies via indirect immunohistochemistry.

## Results

### Patients’ characteristics

In the period from April 2019 to March 2020, 15 adult patients with refractory or relapsed DLBCL were treated with CAR T cell therapy (Tisagenlecleucel). Their median age at CAR T infusion was 59 years (range 31–75 years). Six patients (40%) were female. The number of previous lines of antineoplastic therapies ranged from 2 to 6 (mean: 5). The median time from diagnosis of DLBCL until CAR T infusion was 17 (4–197) months. Patients received 2.3 (0.2–3.5) × 10^8^ CAR T cells (mean, range) after lymphodepletion with Fludarabine and Cyclophosphamide.

### Baseline results

All patients were neurologically examined and received baseline brain MRI, EEG and electrophysiological measurements. Three patients showed mild cognitive impairment (MoCA test < 26 points) at baseline while EEG and imaging findings remained unremarkable. MRI at baseline was inconspicuous in 12/15 cases. In two cases, vascular white matter lesions suggestive of ischemic white matter disease were found. Those lesions went beyond the normal age-related damage and were scored with Fazekas grade 2 [6]. Additionally, one woman exhibited right frontal meningioma as an incidental finding. EEG examinations were normal in 9/15 patients. Subtle vigilance fluctuations (alpha decay) and slight dysrhythmia were observed in individual cases (n = 6). In 11 patients clinical signs and symptoms of polyneuropathy were detectable during neurological examination. Electrophysiological investigations revealed signs of neuropathy in all 15 patients. An association with previous chemotherapies may be suspected.

In 10/15 patients (67%) CSF analysis was performed. No patient showed significant pathological CSF findings: CSF count was normal in all patients (mean 1/μl, range 1–3 cells/μl) (normal value < 5 cells/µl), mean CSF protein was 413 mg/l (range 289–578 mg/l) (normal value ≤ 520 mg/l), and average Qalbumin amounted to 6.48 (range 3.65–9.23) (with the cohort’s average age of 59 years the upper limit of Qalbumin is 7.93). With 9.23 and 8.48, two patients exhibited slightly elevated Qalbumin indicating a subtle disturbance of the blood-CSF barrier function. In no case, oligoclonal bands (type 2 or type 3) as a sign of intrathecal immunoglobulin synthesis became apparent. Likewise, antineuronal antibodies could not be detected in any case. An overview of baseline results is presented in Table 1.

**Table.1:** Baseline characteristics: CSF examination (n = 10) and other diagnostic findings

Diagnostic methods		Patients (n)
Signs and/or symptoms of neuropathy	Clinical	11/15
	Neurography	15/15
Brain MRI	Vascular lesions (Fazekas grade ≥ 2)	2/15
EEG	Subtle vigilance fluctuations	6/15
CSF analysis	Elevated cell count	0/10
	Elevated protein	2/10
	Elevated Qalbumin	2/10
	Oligoclonal bands	0/10

CSF: cerebrospinal fluid; EEG: electroencephalography; MRI: magnetic resonance

### Neurological monitoring during CAR T cell therapy–MoCA testing and ICANS incidence

**Fig. 2:**
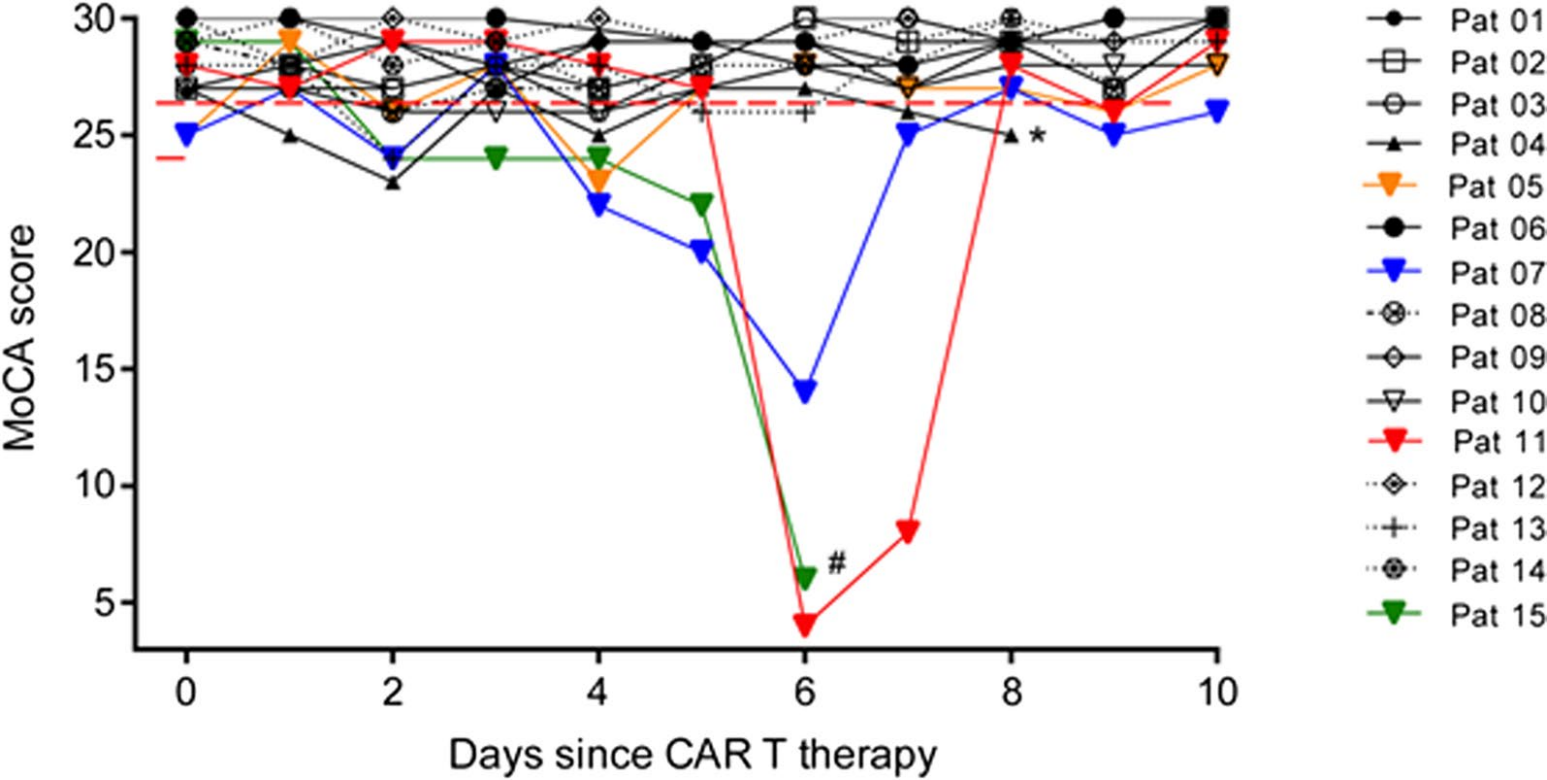
MoCA test results during 10 days monitoring period. All 15 patients were treated with CAR T cell therapy. Except for patient 4 and patient 15 all patients were subjected to a daily MoCA test until the 10th day after therapy. MoCA: Montreal Cognitive Assessment. Pat: patient. *Patient 4 was intubated due to severe CRS on day 8, no further testing; # Patient 15 was transferred to palliative care unit on day 7, no further testing

Patients underwent daily neurological examination and MoCA-testing. The median assessment period was 10 consecutive days after CAR T cell therapy (range 6–14 days) unless cognitive state did not allow further testing. ICANS was diagnosed in 4/15 patients. Conversely, MoCA results remained within the normal range in 11/15 patients and only slight fluctuations were detectable. 3/4 ICANS patients showed a steep drop in MoCA test results on days 4, 5 and 6, respectively (Fig. 2). Especially executive functions, word finding, and retentiveness were disturbed (data not shown). In all three cases, decrease in MoCA testing was followed by further symptoms such as apraxia, tremor, and hallucinations. The patients presented with behavioral changes and seemed suspicious and scared. Symptoms of those three patients with a significant deterioration in MoCA-testing are further on described in more detail. None of the three patients exhibited signs of suggestive of inflammation, particularly no imaging sign suggestive of brain edema on MRI or showed seizures, and thus, they were finally diagnosed with ICANS grade 2. Another patient who reported vivid nightmares and who was slightly disoriented and confused, was diagnosed with ICANS grade 1. She exhibited a MoCA test result of 23/30 points on day 4. Her symptoms spontaneously completely resolved. Two of four ICANS patients required monitoring on ICU. Steroid treatment with intravenous dexamethasone 40 mg per day was initiated in all three cases with ICANS grade 2. The therapy was continued in tapering doses for a total of 4 or 5 days. ICANS symptoms of two patients completely resolved after steroid treatment. One patient showed an improvement, but still had residual neurocognitive symptoms such as orientation and concentration disorder. Interestingly, only one patient presented with CRS before she developed neurological symptoms.

## Description of ICANS°II in case 7

**Fig. 3:**
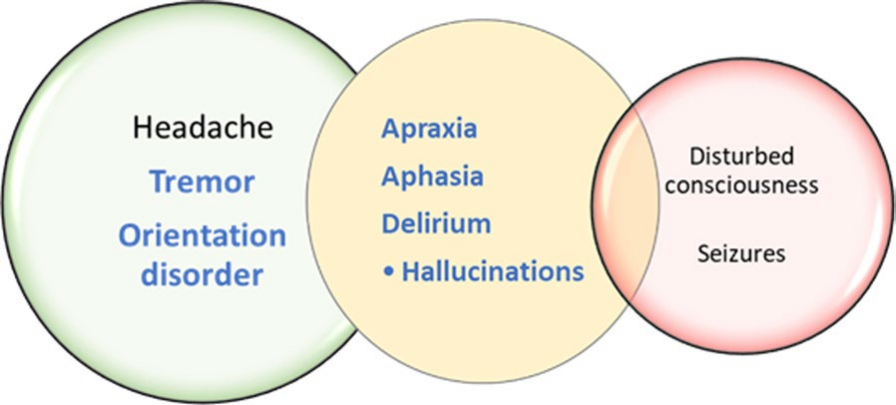
Typical ICANS symptoms independent of ICANS grading. Symptoms occurring in the cases described above are printed in bold and blue. Severity of symptoms increases from left to right

**Fig. 4:**
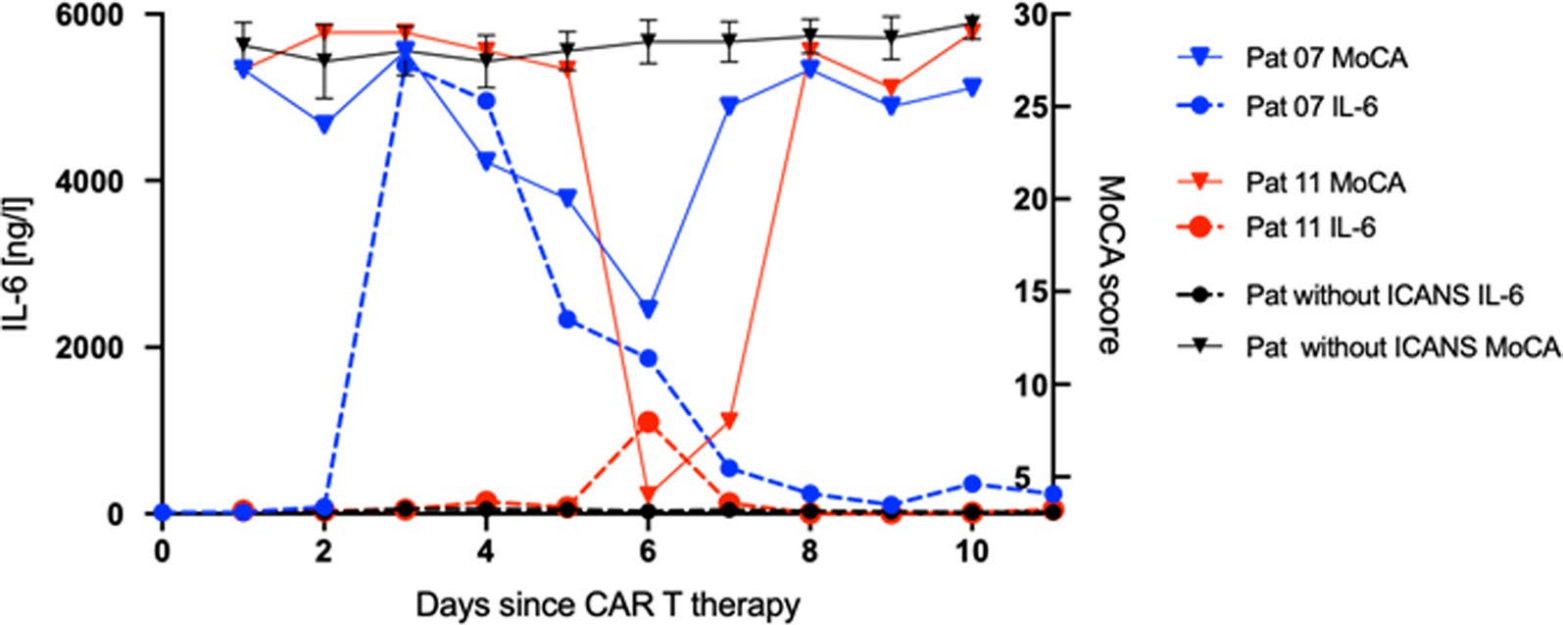
IL-6 levels and MoCA scores of two exemplary ICANS patients compared with all patients that neither had ICANS nor CRS Day 0: day of CAR T cell therapy. Patients 7 and 11 developed immune effector cell-associated neurotoxicity syndrome (ICANS) grade 2. MoCA: Montreal Cognitive Assessment

A 75-year old woman was diagnosed with DLBCL and received R-CHOP (rituximab, cyclophosphamide, doxorubicin, vincristine, prednisone). Disease was progressive 17 months later and she received re-induction therapy with R-DHAP (rituximab, dexamethasone, high dose Ara-C, cisplatin) therapy and high dose BEAM (BCNU, etoposide, Ara-C, melphalane) with autologous stem cell transplant as consolidation therapy. After another relapse, polatuzumab-vedotin in combination with Bendamustin and Rituximab was initiated. Pre-existing conditions included atrial fibrillation, hypothyroidism, and cervical carcinoma in 1985. During baseline examination, a slight cognitive impairment was noted (MoCA test: 25 points). Clinical examination, brain MRI, and EEG did not reveal significant pathological findings. About 2 years after diagnosis of DLBCL, the patient received tisagenlecleucel (cells: 3.4 × 10^8^/kg bodyweight) after lymphodepletion with fludarabine and cyclophophamide. On day 2 after the infusion, she presented with fluid-refractory hypotension and fever. Assuming CRS grade 2 the patient was admitted to the intensive care unit. As IL-6 serum levels rapidly increased on day 3 [5388 ng/l] (Fig. 4) tocilizumab treatment was initiated. On day 4, when IL-6 levels started to slowly decrease [4956 ng/l], first neurocognitive deficits, namely concentration-, memory-, and word finding difficulties could be detected and MoCA-test score was 22/30 points. On day 5 and 6, the score further declined to 20/30 and 14/30 points, respectively. The patient presented with tremor, visual hallucinations, and signs of apraxia and aphasia. Brain MRI showed no pathological findings and consecutively ICANS grade 2 was diagnosed. Dexamethasone was started on day 6. One day after the initiation of dexamethasone treatment (dosage: 40 mg per day), neurological symptoms rapidly improved. On day 8 after CAR T cell infusion, the patient again achieved 28 out of 30 points in the MoCA test and steroid therapy was quickly tapered. Neurological symptoms described are presented in Fig. 3.

### Description of ICANS°II in case 11

A 63-year old man was diagnosed with DLBCL in 2003 and received 6 courses of R-CHOEP (R-CHOP + etoposide) therapy which obtained remission for 13 years. Thirteen years after his initial diagnosis, he suffered from a first relapse and was treated with rituximab and bendamustine. After another relapse and treatment with rituximab, gemcitabine, oxaliplatin for 8 months, decision for CAR T cell therapy was made. The patient’s underlying diseases included terminal renal insufficiency, thrombosis of the right femoral vein, and right hip-total endo prosthesis. Except for vascular white matter changes on brain MRI and clinical and electrophysiological signs of polyneuropathy, neurological baseline remained inconspicuous. He received fludarabine and cyclophosphamide for lymphodepletion and subsequently tisagenlecleucel (cells: 3.2 × 10^8^/kg bodyweight). On day 2 after the infusion, neutropenic fever was diagnosed but no other signs of CRS became obvious. Six days after CAR T cell therapy the patient suddenly developed visual hallucinations, apraxia, aphasia, and orientation disorder. MoCA test result deteriorated from normal values to only 4 of 30 points. ICANS grade 2 was diagnosed as brain MRI showed no signs of edema or other pathologies. He was transferred to the intensive care unit and was treated with dexamethasone (initial dosage: 40 mg per day) in descending doses for 5 days. His symptoms improved within 2 days (28/30 points at MoCA on day 8). No residual neurocognitive deficits were detectable. Neurological symptoms described are presented in Fig. 3.

### Description of ICANS°II in case 15

A 62-year old man developed secondary DLBCL 4 months after the initial diagnosis of follicular lymphoma. He initially received 4 courses of obinutuzumab-bendamustine and further 6 courses of R-CHOP + rituximab and methotrexate as disease was progressive. After a second progress, the patient obtained R-DHAP (1 course) and R-ICE (rituximab, ifosfamide, carboplatin, etoposide). Due to further disease progression, CAR T cell therapy was initiated 10 months after the diagnosis of DLBCL. Regarding other underlying diseases, the patient was known to have hypercholesterolemia, hypothyroidism and history of postrenal acute kidney failure. Except for clinical and electrophysiological signs of polyneuropathy, neurological baseline examination was inconspicuous. He received fludarabine and cyclophosphamide for lymphodepletion followed by tisagenlecleucel (cells: 0.2 × 10^8^/kg bodyweight). After the CAR T cell infusion, an increasing hypercalcemia occurred which made an emergency dialysis necessary on day 5 after CAR T cell therapy. A tumor progression was assumed to be the underlying cause of hypercalcemia. MoCA test results were already below the limit value of 26 points from day 2, but on day 6 there was a considerable drop to only 6 points (Fig. 2). The patient presented with agitation, apraxia and orientation disorder. Assuming ICANS grade 2, he was treated with dexamethasone (initial dosage: 40 mg per day) in descending doses for 5 days. Neurological deficits improved, but orientation and concentration deficits were still present. Due to tumor progression despite CAR T cell therapy, the patient was finally transferred to the palliative care unit on day 7 after the CAR T cell infusion.

### Comparison of baseline characteristics and diagnostic findings of patients with and without ICANS

The four patients who developed ICANS have been compared with those who showed no signs of neurotoxicity regarding baseline diagnostic criteria (Table 2). ICANS patients did not show a significant difference regarding age compared with non-ICANS patients. No significant difference was observed in baseline CSF cell count, blood/CSF barrier disturbance, and presence vascular white matter lesions on brain MRI. The groups also did not differ with regard to previous vascular diseases, tumor burden (measured by LDH value), and platelet count before CAR-T cell therapy.

**Table.2:** Comparison of baseline parameters of patients with ICANS and patients without

	ICANS (N = 4)	No ICANS (N = 11)	*p*-value
Age (mean ± SD)	63 ± 10.3	54 ± 14.5	0.22
CSF cell count (mean ± SD)	1.8 ± 2.1	0.89 ± 0.74	0.65
Blood/CSF barrier disturbance	1/3	1/7	0.64
MRI vascular lesions (number)	2/4	3/11	0.51
Vascular comorbidities (number)	1.25 ± 0.96	1.0 ± 1.56	0.72
Thrombocytes prior to CAR T therapy (/µl)	142,000 ± 137,117	68,545 ± 44,408	0.37
LDH prior to CAR T therapy (U/l)	573 ± 566	333 ± 167	0.46

Data presented as means ± SD. CRS: cytokine release syndrome; CSF: cerebrospinal fluid; ICANS: immune effector cell associated neurotoxicity syndrome; LDH: lactate dehydrogenase; MRI: magnetic resonance imaging; Student's two-tailed t test was used to calculate the *p*-values

### Decrease in MoCA testing is associated with increased serum IL-6 levels in two patients with ICANS grade 2

MoCA results and IL-6 serum levels were compared in two ICANS grade 2 patients who were observed until the end of the follow-up period. In patient 7, a steep increase in IL-6 levels from day 2 to day 3 was detected (Fig. 4) which coincided with the onset of CRS symptoms. Thus, treatment with tocilizumab from day 2 was started (four doses in total). The deterioration in MoCA testing started with a slight delay on day 4 and reached its lowest point on day 6 when steroid treatment was initiated. Afterwards, cognitive functions rapidly improved and MoCA results normalized on day 8. IL-6 values already decreased on day 5 and almost reached their initial level on day 9. Patient 11 who showed no signs of CRS, presented with an IL-6 serum peak of 1103 ng/l on day 6. The significant deterioration in MoCA testing occurred simultaneously with the IL-6 increase and intravenous steroid treatment was started. While IL-6 values almost normalized the next day, it took 1 day longer for the neurocognitive impairment to normalize. Compared to those ICANS patients, all other patients who did not show any neurocognitive abnormalities exhibited only slight fluctuations in IL-6 levels but no significant increases. Patient 4 who had a fulminant CRS must be mentioned as an exception. Figure 4 demonstrates the IL-6 course and MoCA-score development of all non-ICANS patients.

## Discussion

Within our cohort, 15 patients have been treated with CAR T cell therapy. Besides one patient with only subtle neurological symptoms categorized as ICANS grade 1, three other patients developed more distinct neurological deficits and were diagnosed with ICANS grade 2. They presented with similar symptoms such as tremor, orientation disorder, apraxia, aphasia, and hallucinations. Additionally, all ICANS patients with grade 2 showed behavioral changes and anxiety. The timing of symptom development (about 5 days after therapy onset), the combination of symptoms and the sufficient response to steroid therapy was common to all three patients. The similarity may be an indication that ICANS is a very specific side effect and not comparable to other disorders such as septic encephalopathy. It should be noted that the incidence of ICANS in our local German cohort is lower than in other studies that have been published so far. However, it must be mentioned that the different CAR T products vary significantly with respect to the occurrence of higher-grade neurotoxicity. In the tisagenlecleucel pivotal study, grade 3 and 4 neurotoxicities occurred in 12% of patients [23], whereas this rate was 28% in the ZUMA-1 study on axicabtagene ciloleucel (axi-cel) [17]. A univariate analysis showed that younger age, previous neurological conditions such as epilepsy or history of headache, toxicity after previous methotrexate therapy, high tumor burden and high CAR T cell dose might increase the risk of neurotoxicity. Patients with grade 3 or 4 neurotoxicity showed earlier and higher fever, more severe hypotension, coagulation disorders and a capillary leak, which corresponds to the effects of vascular dysfunction in CRS. These results have been similarly observed in other studies [15, 22, 24]. In contrast to the previous studies, the four ICANS patients in our cohort were not younger than patients without neurotoxicity. Patients who developed neurological symptoms did not suffer more frequently from concomitant vascular diseases and also did not show vascular lesions more frequently on brain MRI. Furthermore, no significant differences were observed regarding baseline LDH values, baseline platelets, and baseline CSF results. Detailed baseline examination including neurography revealed polyneuropathy in all patients of our cohort. Toxic damage from the previous multiple chemotherapies can be assumed.

Regarding cell and molecular levels, monocytes and macrophages as well as their cytokines IL-1 and IL-6 were shown to play a crucial role in the development of CRS and neurotoxicity in animal models. Analyses of the cytokine profiles in patients undergoing CAR T cell therapy have identified several molecules such as IL-6 that were associated with the occurrence of neurotoxicity [9, 17, 22]. In our cohort, increased IL-6 levels were found during ICANS development independent of the occurrence of a CRS. IL-6 blockade with tocilizumab has been proven to be successful in CRS [12]. However, tocilizumab has not been shown to reduce the severity of neurotoxicity or to minimize neurotoxicity-associated morbidity and mortality. In contrast, it was suggested that peripheral IL-6 receptor blockade may lead to increased circulation of IL-6 into the CNS and thus to exacerbation of neurotoxicity [4, 19]. Other therapeutic considerations are based on the fact that destruction of the endothelium or rather the blood–brain barrier seems to be crucial for the development of neurotoxicity. Therefore, treatment with steroids and symptomatic therapy are preferentially used to combat CAR T cell associated neurotoxicity.

Our results show that a structured neurological examination and monitoring leads to a profound diagnosis and effective treatment of ICANS using dexamethasone, consecutively resulting in an excellent neurological outcome. The first 10 days of monitoring after CAR T administration are of particular importance, since CRS usually manifests during the first week (peak 1–3 days) and ICANS is most likely to occur on days 5–8 (our cohort and [21]). Neurological co-supervision is obligatory and is required by the manufacturers of CAR T cell products due to the requirements of regulatory authorities. Before initiating the therapy, a detailed clinical neurological examination is recommended to identify possible neurological symptoms independent of treatment. On the day of the infusion as well as on the following 10 days, daily clinical examinations should be performed in order to quickly recognize a potentially fatal neurotoxicity. Especially in patients with increasing IL-6 serum concentrations, the MoCA-test is a very sensitive tool to detect incipient neurotoxicity. In addition, we recommend to perform an MRI of the brain prior to therapy and in case of occurrence of neurological side effects, as well as in the absence of improvement of side effects due to steroid therapy, even though in the cases described here the MRI showed no abnormalities.

The observation that no patient developed higher grade neurotoxicity in our cohort might be due to the fact that ICANS was recognized when the first neurological symptoms developed and that therapy with dexamethasone was therefore started relatively early. On the other hand, the rate of grade 3 and 4 neurotoxicity with tisagenlecleucel is comparatively low, even in larger studies [23]. We can only speculate about the low incidence of overall neurotoxicity in our cohort. So far, studies have been published mainly from North America. Patient selection and differences in potential risk factors might be suggested. The comparison of the different patient groups should be subject of further research.

The main limitation of the study is that the small cohort size naturally limits comparability with larger studies such as the pivotal study for tisagenlecleucel, JULIET. Concluding causal relationships is problematic given the small number of patients. In addition, only patients treated with tisagenlecleucel were included. Axicabtagene ciloleucel (axi-cel), which showed a higher neurotoxicity rate in the pivotal study, was not used here. Nevertheless, this work emphasizes the importance of thorough neurological monitoring and suggests resorting to the more sensitive MoCA-test, particularly in patients with rapid IL-6 increases.

## Conclusions

In conclusion, the incidence of ICANS was low in our local German cohort and occurred in only every fifth patient. Furthermore, only low-grade neurotoxicity was found during daily examination. Whether the rate of patients with neurotoxicity was so low because symptoms were recognized and treated early or because neurotoxicity is quite rare in tisagenlecleucel patients anyway remains an open question. We conclude that a structured neurological baseline examination and close neurological monitoring is of great importance when using CAR T cell products. Neurological side effects can have potentially fatal consequences and should be detected and treated as early as possible.

## Supplementary Information

**Additional file 1.** Characteristics of all individual patients within the cohort. Patients’ characteristics. COPD: chronic obstructive pulmonary disease; DLBCL: diffuse large B cell lymphoma; DM: diabetes mellitus; f: female; FL: follicular lymphoma; h/o: history of; IPI: international prognostic index; m: male; MoCA: Montreal Cognitive Assessment; r/r: relapsing/refractory; SCT: stem cell transplant.

**Additional file 2.** Baseline neurological examination results and CRS/ICANS incidence. CRS: cytokine release syndrome; ICANS: immune effector cell associated neurotoxicity syndrome; ICU: intensive care unit; MoCA: Montreal Cognitive Assessment; n/a: not available.

## Data Availability

All data and material are provided by the corresponding author on reasonable request. The datasets supporting the conclusions of this article are included within the article and its additional files.

## Abbreviations

ALL: Acute lymphoblastic leukemia
BEAM: Bis-chlorethyl-nitroso-urea, etoposide, Ara-C, melphalane
CAR: Chimeric antigen receptor
CR: Complete remission
CRS: Cytokine release syndrome
CSF: Cerebrospinal fluid
DWI: Diffusion-weighted imaging
EEG: Electroencephalography
ENG: Electroneurography
FLAIR: Fluid attenuated inversion recovery
ICANS: Immune effector cell-associated neurotoxicity syndrome
MoCA: Montreal Cognitive Assessment
MRI: Magnetic resonance imaging
OCB: Oligoclonal bands
ORR: Objective response rate
R-CHOP: Rituximab, cyclophosphamide, doxorubicin, vincristine, prednisone
R-DHAP: Rituximab, dexamethasone, high dose Ara-C, cisplatin
R-ICE: Rituximab, ifosfamide, carboplatin, etoposide
r/rDLBCL: Relapsing or refractory diffuse large B cell lymphoma
SWI: Susceptibility weighted imaging

## References

[1] Brooks S, Frey N, Porter D, June C, Lacey S, Bagg A (2016). The cytological features of CAR(T) cells. British Journal of Haematology.

[2] Brudno JN, Kochenderfer JN (2016). Toxicities of chimeric antigen receptor T cells: Recognition and management. Blood.

[3] Brudno JN, Kochenderfer JN (2018). Chimeric antigen receptor T-cell therapies for lymphoma. Nature Reviews Clinical Oncology.

[4] Chen F, Teachey DT, Pequignot E, Frey N, Porter D, Maude SL, Grupp SA, June CH, Melenhorst JJ, Lacey SF (2016). Measuring IL-6 and sIL-6R in serum from patients treated with tocilizumab and/or siltuximab following CAR T cell therapy. Journal of Immunological Methods.

[5] Crump M, Neelapu SS, Farooq U, Van Den Neste E, Kuruvilla J, Ahmed MA, Link BK, Hay AE, Cerhan JR, Zhu L, Boussetta S (2017). Outcomes in refractory diffuse large B-cell lymphoma: Results from the international SCHOLAR-1 study. Blood.

[6] Fazekas F, Chawluk JB, Alavi A, Hurtig HI, Zimmerman RA (1987). MR signal abnormalities at 1.5 T in Alzheimer's dementia and normal aging. American Journal of Roentgenology.

[7] Fry TJ, Shah NN, Orentas RJ, Stetler-Stevenson M, Yuan CM, Ramakrishna S, Wolters P, Martin S, Delbrook C, Yates B, Shalabi H, Fountaine TJ, Shern JF, Majzner RG, Stroncek DF, Sabatino M, Feng Y, Dimitrov DS, Zhang L, Nguyen S, Qin H, Dropulic B, Lee DW, Mackall CL (2018). CD22-targeted CAR T cells induce remission in B-ALL that is naive or resistant to CD19-targeted CAR immunotherapy. Nature Medicine.

[8] Gust J, Hay KA, Hanafi L-A, Li D, Myerson D, Gonzalez-Cuyar LF, Yeung C, Liles WC, Wurfel M, Lopez JA, Chen J, Chung D, Harju-Baker S, Özpolat T, Fink KR, Riddell SR, Maloney DG, Turtle CJ (2017). Endothelial activation and blood-brain barrier disruption in neurotoxicity after adoptive immunotherapy with CD19 CAR-T cells. Cancer Discovery.

[9] Hay KA, Hanafi L-A, Li D, Gust J, Liles WC, Wurfel MM, López JA, Chen J, Chung D, Harju-Baker S, Cherian S, Chen X, Riddell SR, Maloney DG, Turtle CJ (2017). Kinetics and biomarkers of severe cytokine release syndrome after CD19 chimeric antigen receptor-modified T-cell therapy. Blood.

[10] Hümmert MW, Wurster U, Bönig L, Schwenkenbecher P, Sühs K-W, Alvermann S, Gingele S, Skripuletz T, Stangel M (2019). Investigation of oligoclonal IgG bands in tear fluid of multiple sclerosis patients. Frontiers in Immunology.

[11] Klem GH, Luders HO, Jasper HH, Elger C (1999). The ten-twenty electrode system of the International Federation. The International Federation of Clinical Neurophysiology. Electroencephalography and Clinical Neurophysiology Supplement.

[12] Le RQ, Li L, Yuan W, Shord SS, Nie L, Habtemariam BA, Przepiorka D, Farrell AT, Pazdurb R (2018). FDA approval summary: Tocilizumab for treatment of chimeric antigen receptor t cell-induced severe or life-threatening cytokine release syndrome. The Oncologist.

[13] Lee DW, Santomasso BD, Locke FL, Ghobadi A, Turtle CJ, Brudno JN, Maus MV, Park JH, Mead E, Pavletic S, Go WY, Eldjerou L, Gardner RA, Frey N, Curran KJ, Peggs K, Pasquini M, DiPersio JF, van den Brink MRM, Komanduri KV, Grupp SA, Neelapu SS (2019). ASTCT consensus grading for cytokine release syndrome and neurologic toxicity associated with immune effector cells. Biology of Blood and Marrow Transplantation.

[14] Locke FL, Neelapu SS, Bartlett NL, Siddiqi T, Chavez JC, Hosing CM, Ghobadi A, Budde LE, Bot A, Rossi JM, Jiang Y, Xue AX, Elias M, Aycock J, Wiezorek J, Go WY (2017). Phase 1 results of ZUMA-1: A multicenter study of KTE-C19 anti-CD19 CAR T cell therapy in refractory aggressive lymphoma. Molecular Therapy.

[15] Maude SL, Frey N, Shaw PA, Aplenc R, Barrett DM, Bunin NJ, Chew A, Gonzalez VE, Zheng Z, Lacey SF, Mahnke YD, Melenhorst JJ, Rheingold SR, Shen A, Teachey DT, Levine BL, June CH, Porter DL, Grupp SA (2014). Chimeric antigen receptor T cells for sustained remissions in leukemia. New England Journal of Medicine.

[16] Maude SL, Laetsch TW, Buechner J, Rives S, Boyer M, Bittencourt H, Bader P, Verneris MR, Stefanski HE, Myers GD, Qayed M, De Moerloose B, Hiramatsu H, Schlis K, Davis KL, Martin PL, Nemecek ER, Yanik GA, Peters C, Baruchel A, Boissel N, Mechinaud F, Balduzzi A, Krueger J, June CH, Levine BL, Wood P, Taran T, Leung M, Mueller KT, Zhang Y, Sen K, Lebwohl D, Pulsipher MA, Grupp SA (2018). Tisagenlecleucel in children and young adults with B-cell lymphoblastic leukemia. New England Journal of Medicine.

[17] Neelapu SS, Locke FL, Bartlett NL, Lekakis LJ, Miklos DB, Jacobson CA, Braunschweig I, Oluwole OO, Siddiqi T, Lin Y, Timmerman JM, Stiff PJ, Friedberg JW, Flinn IW, Goy A, Hill BT, Smith MR, Deol A, Farooq U, McSweeney P, Munoz J, Avivi I, Castro JE, Westin JR, Chavez JC, Ghobadi A, Komanduri KV, Levy R, Jacobsen ED, Witzig TE, Reagan P, Bot A, Rossi J, Navale L, Jiang Y, Aycock J, Elias M, Chang D, Wiezorek J, Go WY (2017). Axicabtagene ciloleucel CAR T-cell therapy in refractory large B-cell lymphoma. New England Journal of Medicine.

[18] Neelapu SS, Tummala S, Kebriaei P, Wierda W, Gutierrez C, Locke FL, Komanduri KV, Lin Y, Jain N, Daver N, Westin J, Gulbis AM, Loghin ME, de Groot JF, Adkins S, Davis SE, Rezvani K, Hwu P, Shpall EJ (2018). Chimeric antigen receptor T-cell therapy—assessment and management of toxicities. Nature Reviews. Clinical Oncology.

[19] Nishimoto N, Terao K, Mima T, Nakahara H, Takagi N, Kakehi T (2008). Mechanisms and pathologic significances in increase in serum interleukin-6 (IL-6) and soluble IL-6 receptor after administration of an anti-IL-6 receptor antibody, tocilizumab, in patients with rheumatoid arthritis and Castleman disease. Blood.

[20] Porter D, Frey N, Wood PA, Weng Y, Grupp SA (2018). Grading of cytokine release syndrome associated with the CAR T cell therapy tisagenlecleucel. Journal of Hematology & Oncology.

[21] Rubin DB, Danish HH, Ali AB, Li K, LaRose S, Monk AD, Cote DJ, Spendley L, Kim AH, Robertson MS, Torre M, Smith TR, Izzy S, Jacobson CA, Lee JW, Vaitkevicius H (2019). Neurological toxicities associated with chimeric antigen receptor T-cell therapy. Brain.

[22] Santomasso BD, Park JH, Salloum D, Riviere I, Flynn J, Mead E, Halton E, Wang X, Senechal B, Purdon T, Cross JR, Liu H, Vachha B, Chen X, DeAngelis LM, Li D, Bernal Y, Gonen M, Wendel H-G, Sadelain M, Brentjens RJ (2018). Clinical and biological correlates of neurotoxicity associated with CAR T-cell therapy in patients with B-cell acute lymphoblastic leukemia. Cancer Discovery.

[23] Schuster SJ, Bishop MR, Tam CS, Waller EK, Borchmann P, McGuirk JP, Jäger U, Jaglowski S, Andreadis C, Westin JR, Fleury I, Bachanova V, Foley SR, Ho PJ, Mielke S, Magenau JM, Holte H, Pantano S, Pacaud LB, Awasthi R, Chu J, Anak Ö, Salles G, Maziarz RT, for the JULIET Investigators (2019). Tisagenlecleucel in adult relapsed or refractory diffuse large B-cell lymphoma. New England Journal of Medicine.

[24] Schuster SJ, Svoboda J, Chong EA, Nasta SD, Mato AR, Anak Ö, Brogdon JL, Pruteanu-Malinici I, Bhoj V, Landsburg D, Wasik M, Levine BL, Lacey SF, Melenhorst JJ, Porter DL, June CH (2017). Chimeric antigen receptor T cells in refractory B-cell lymphomas. New England Journal of Medicine.

[25] Schwenkenbecher P, Sarikidi A, Wurster U, Bronzlik P, Sühs KW, Raab P, Stangel M, Pul R, Skripuletz T (2016). McDonald criteria 2010 and 2005 compared: Persistence of high oligoclonal band prevalence despite almost doubled diagnostic sensitivity. International Journal of Molecular Sciences.

[26] Stahl K, Schmidt BM, Hoeper MM, Skripuletz T, Möhn N, Beutel G, Eder M, Welte T, Ganser A, Falk CS, Koenecke C (2020). Extracorporeal cytokine removal in severe CAR-T cell associated cytokine release syndrome. Journal of Critical Care.

[27] Suhs KW, Wegner F, Skripuletz T, Trebst C, Tayeb SB, Raab P, Stangel M (2015). Heterogeneity of clinical features and corresponding antibodies in seven patients with anti-NMDA receptor encephalitis. Experimental and Therapeutic Medicine.

[28] Titov A, Petukhov A, Staliarova A, Motorin D, Bulatov E, Shuvalov O, Soond SM, Piacentini M, Melino G, Zaritskey A, Barlev NA (2018). The biological basis and clinical symptoms of CAR-T therapy-associated toxicites. Cell Death & Disease.

