# Supplementary material for: Neurological management and work-up of neurotoxicity associated with CAR T cell therapy
Source: Neurol Res Pract. 2022 Jan 10;4:1. doi: 10.1186/s42466-021-00166-5 (PMC8744256; doi:10.1186/s42466-021-00166-5)
Supplement: Supplementary file 1 — Additional file 1. Characteristics of all individual patients within the cohort. Patients’ characteristics. COPD: chronic obstructive pulmonary disease; DLBCL: diffuse large B cell lymphoma; DM: diabetes mellitus; f: female; FL: follicular lymphoma; h/o: history of; IPI: international prognostic index; m: male; MoCA: Montreal Cognitive Assessment; r/r: relapsing/refractory; SCT: stem cell transplant. [file 42466_2021_166_MOESM1_ESM.docx]

| CART-ID | Gender | Age at CART (years) | Diagnosis | | Stadium | | IPI | Previous lines of antineoplastic therapy (#) | Previous autologous SCT | Dose reduced lymphodepletion | Time from Diagnosis to CART (months) | CART cell dose (x10^8 /kg BW) | Preexisting Conditions |
| --- | --- | --- | --- | --- | --- | --- | --- | --- | --- | --- | --- | --- | --- |
| 001 | f | 55 | transformed FL | | IVB | | 2 | 5 | no | no | 4 | 1.2 | Chronic kidney disease (G3a) |
| 002 | m | 60 | transformed FL | | IVB | | 2 | 3 | no | no | 17 | 2.0 |  |
| 003 | m | 59 | DLBCL | | IA | | 0 | 5 | yes | no | 81 | 2.5 | Obesity, polyneuropathy, migraine |
| 004 | m | 64 | DLBCL | | IIIA | | 1 | 5 | yes | yes | 175 | 2.5 |  |
| 005 | f | 50 | transformed FL (leg-type) | | IIIA | | 2 | 6 | no | no | 16 | 2.77 | Peroneal nerve paralysis (l), deep vein thrombosis, β thalassemia minor |
| 006 | f | 31 | DLBCL | | IIE | | 1 | 5 | no | no | 21 | 1.7 |  |
| 007 | f | 74 | DLBCL | | IIIA | | 2 | 5 | yes | no | 25 | 3.4 | Atrial fibrillation, hypothyroidism, h/o pulmonary embolism, h/o cervical carcinoma |
| 008 | m | 36 | transformed FL (Burkitt-like) | | IIA | | 0 | 4 | no | no | 11 | 3.5 | Arterial hypertension, deep vein thrombosis |
| 009 | f | 59 | DLBCL | | IIIB | | 2 | 4 | yes | no | 20 | 1.0 | H/o pulmonary embolism |
| 010 | m | 66 | DLBCL | | IVA | | 2 | 4 | yes | no | 53 | 2.8 | Multinodular goiter, h/o hepatitis B |
| 011 | m | 65 | DLBCL | | IVA | | 1 | 3 | no | yes | 197 | 3.2 | Chronic kidney disease (G5), deep vein thrombosis |
| 012 | m | 32 | DLBCL | | IIA | | 0 | 2 | no | no | 12 | 3.2 | none |
| 013 | m | 75 | DLBCL | | IVA | | 2 | 5 | no | no | 15 | 2.3 |  |
| 014 | f | 56 | | DLBCL | | IIE | 1 | 4 | yes | no | 10 | 2.1 |  |
| 015 | m | 62 | | transformed FL | | IVA | 4 | 5 | no | yes | 10 | 0.2 | Hypercholesterolemia, hypothyroidism, h/o postrenal acute kidney injury (ureteral stents) |

**Additional file 1**: Patients’ characteristics. COPD: chronic obstructive pulmonary disease; DLBCL: diffuse large B cell lymphoma; DM: diabetes mellitus; f: female; FL: follicular lymphoma; h/o: history of; IPI: international prognostic index; m: male; MoCA: Montreal Cognitive Assessment; r/r: relapsing/refractory; SCT: stem cell transplant
