# Supplementary material for: Neurological management and work-up of neurotoxicity associated with CAR T cell therapy
Source: Neurol Res Pract. 2022 Jan 10;4:1. doi: 10.1186/s42466-021-00166-5 (PMC8744256; doi:10.1186/s42466-021-00166-5)
Supplement: Supplementary file 2 — Additional file 2. Baseline neurological examination results and CRS/ICANS incidence. CRS: cytokine release syndrome; ICANS: immune effector cell associated neurotoxicity syndrome; ICU: intensive care unit; MoCA: Montreal Cognitive Assessment; n/a: not available. [file 42466_2021_166_MOESM2_ESM.docx]

| CART-ID | Neurological examination | MoCA baseline (points out of 30) | CRS  (grade) | ICANS  (grade) | CRS/ICANS  treatment |
| --- | --- | --- | --- | --- | --- |
| 001 | pallhypaesthesia, otherwise no deficits | 30 | II | n/a | ICU |
| 002 | pallhypaesthesia, otherwise no deficits | 27 | n/a | n/a |  |
| 003 | signs of neuropathy, otherwise no deficits | 28 | n/a | n/a | n/a |
| 004 | pallhypaesthesia, slight gait disorder | 27 | IV | n/a | ICU, Tocilizumab, CytoSorb© |
| 005 | paresis of the left leg | 25 | n/a | I | n/a |
| 006 | left-sided peroneal lesion, tingling paresthesia | 30 | n/a | n/a | n/a |
| 007 | tingling paresthesia, otherwise no deficits | 25 | II | II | ICU, Tocilizumab, Steroids |
| 008 | inconspicuous | 29 | I | n/a | n/a |
| 009 | pallhypaesthesia, otherwise no deficits | 27 | n/a | n/a | n/a |
| 010 | inconspicuous | 23 | n/a | n/a | n/a |
| 011 | proximally emphasized right leg paresis, signs of neuropathy | 28 | n/a | II | ICU, Steroids |
| 012 | inconspicuous | 30 | I | n/a | Monitoring |
| 013 | lower limb ataxia, lower limb pallanesthesia | 28 | n/a | n/a |  |
| 014 | pallhypaesthesia, weak reflexes, otherwise no deficits | 29 | n/a | n/a | n/a |
| 015 | pallanesthesia and weak reflexes of lower extremities | 29 | n/a | II | Steroids |

**Additional file 2**: Baseline neurological examination results and CRS/ICANS incidence. CRS: cytokine release syndrome; ICANS: immune effector cell associated neurotoxicity syndrome; ICU: intensive care unit; MoCA: Montreal Cognitive Assessment; n/a: not available
